# Interventions to prevent hemodynamic instability during renal replacement therapy for acute kidney injury: a systematic review protocol

**DOI:** 10.1186/s13643-017-0512-9

**Published:** 2017-06-15

**Authors:** Adrianna Douvris, Swapnil Hiremath, Lauralyn McIntyre, Lindsey Sikora, Catherine Weber, Edward G. Clark

**Affiliations:** 1 Department of Medicine, University of Ottawa, Ottawa, Ontario Canada; 2 Division of Nephrology, Department of Medicine and Kidney Research Centre, Ottawa Hospital Research Institute, University of Ottawa, Ottawa, Canada; 3 Division of Critical Care, Department of Medicine, The Ottawa Hospital, Ottawa, Ontario Canada; 4 Centre for Transfusion Research, Clinical Epidemiology Program, Ottawa Hospital Research Institute, Ottawa, Ontario Canada; 5 Health Sciences Library, University of Ottawa, Ottawa, Ontario Canada; 6 Division of Nephrology, McGill University Health Centre, Montreal, Quebec Canada; 7 The Ottawa Hospital – Riverside Campus, 1967 Riverside Drive, Ottawa, Ontario K1H 7W9 Canada

**Keywords:** Acute kidney injury, Renal replacement therapy, Intradialytic hypotension, Trials, Patients, Blood pressure, Dialysis

## Abstract

**Background:**

Hemodynamic instability during renal replacement therapy (HIRRT) in the form of intradialytic hypotension (IDH) is a frequent complication of hemodialysis in end-stage kidney disease (ESKD), and most studies have focused on this chronic population. However, HIRRT is also an important concern for critically ill ICU patients with acute kidney injury (AKI), complicating an estimated 30% of dialysis treatments in this population. HIRRT can exacerbate organ hypoperfusion in the setting of critical illness and may negatively impact renal recovery in the AKI population. This is a protocol for a systematic review to synthesize the evidence surrounding dialysis-related interventions used to minimize HIRRT in critically ill patients with RRT-requiring AKI. This protocol has been registered with the International Prospective Register of Systematic Reviews (PROSPERO) database.

**Methods/design:**

We will search MEDLINE, EMBASE, and CENTRAL databases in collaboration with a health information specialist using a comprehensive search strategy. We will also supplement our search with a scan of the “gray literature” to identify relevant ongoing trials or conference abstracts. Observational studies and clinical trials will be included in our analysis. Our outcomes will include the incidence of HIRRT, dialysis-related complications, in-hospital mortality, and renal recovery. Prior to our search, we performed an initial search of these databases and PROSPERO, which yielded no prior or ongoing systematic reviews on this topic. Two reviewers will independently screen the list of identified abstracts using pre-defined inclusion and exclusion criteria. Two reviewers will then independently extract data from selected studies and undertake an assessment of their quality using validated tools.

**Discussion:**

HIRRT is a common complication of renal replacement therapy not only in ESKD but also in the critically ill AKI population. It can result in early discontinuation of dialysis, further organ injury from hypoperfusion, and possibly negatively impact renal recovery. This systematic review will synthesize the existing evidence on the interventions employed to predict or prevent episodes HIRRT in critically ill patients with RRT-requiring AKI. This systematic review will allow for an understanding the current evidence for interventions to limit HIRRT in AKI and, in doing so, may also highlight areas in need of further research.

**Systematic review registration:**

PROSPERO CRD42016037754

**Electronic supplementary material:**

The online version of this article (doi:10.1186/s13643-017-0512-9) contains supplementary material, which is available to authorized users.

## Background

Episodes of hemodynamic instability during renal replacement therapy (HIRRT) in the form of intradialytic hypotension (IDH) frequently complicates maintenance hemodialysis treatments in end-stage kidney disease (ESKD) patients [1]. Minor consequences of this form of HIRRT include symptoms such as post-dialysis fatigue [1, 2]. More serious complications include bowel ischemia, myocardial infarction, and access thrombosis [2]. Recent studies have highlighted that myocardial stunning [3] and brain white-matter changes [4] are also associated with HIRRT in the form of IDH. Furthermore, the occurrence of HIRRT can limit delivery of adequate dialysis treatment as interventions frequently undertaken in response to HIRRT include terminating dialysis sessions early or reducing ultrafiltration goals [1, 2]. Lastly, in maintenance hemodialysis patients, more frequent episodes of HIRRT (as IDH) may result in more rapid loss of residual renal function over time [5, 6].

Most studies of interventions to prevent or limit HIRRT have focused specifically on IDH in maintenance hemodialysis patients with ESKD; however, IDH is only one form of HIRRT which is also an important concern amongst critically ill patients who require hemodialysis or other forms of RRT (such as slow low-efficiency dialysis (SLED) or continuous renal replacement therapy (CRRT)) in the context of acute kidney injury (AKI). AKI that necessitates RRT is a frequent and serious complication of critical illness with an incidence of 2 to 3% amongst all patients admitted to the intensive care unit (ICU) [7]. One study suggests that HIRRT complicates an estimated 30% of hemodialysis treatments for AKI patients in the ICU [8]. This is particularly important since there is some evidence to suggest that HIRRT negatively impacts renal recovery in AKI [9], possibly similar to how HIRRT (in the form of IDH) accelerates the loss of residual kidney function in those on maintenance hemodialysis [5, 6].

The goal of this study is to summarize all observational and trial evidence related to interventions to prevent or limit HIRRT in AKI patients. This encompasses AKI that is treated with the most commonly utilized RRT modalities in the setting of critical illness (intermittent hemodialysis, SLED, and CRRT).

## Objectives

The primary objective of this systematic review is to synthesize the evidence surrounding interventions to limit HIRRT in critically ill patients with AKI. In particular, we wish to assess the impact of various dialysis-related interventions that have been studied to minimize its occurrence.

### Free form question

- What is the effectiveness of interventions or strategies employed to limit HIRRT in AKI?

## Methods/design

This protocol is reported in accordance with the PRISMA-P 2015 checklist [10] (see Additional file 1).

### Criteria for considering studies for this review

The planned systematic review and meta-analysis will be performed and reported as proscribed by the Preferred Reporting Items for Systematic Reviews and Meta-Analyses (PRISMA) statement [11]. This protocol was previously registered with the International Prospective Register of Systematic Reviews (PROSPERO): CRD42016037754.

#### Types of studies

All studies that assess a dialysis-related intervention to limit or prevent HIRRT in AKI populations, including interventional studies (including cross-over, parallel arm, or cluster trials) and observational (including cohort, cross-sectional, retrospective, prospective, or combined) studies, will be eligible. In addition, to be eligible for inclusion, studies must meet the following criteria.

#### Population

Studies with critically ill adult (18 years of age or older) patients with AKI treated with RRT (including intermittent hemodialysis, SLED, or CRRT but excluding those treated with peritoneal dialysis in this context) will be included.

#### Exposure/intervention

Studies with a dialysis-related intervention (or a modifiable factor) related to the application of RRT (e.g., dialysate temperature, ultrafiltration method, dialysate electrolyte concentration, sodium profiling, ultrafiltration profiling, medications to prevent or limit HIRRT) will be included. (Observational studies that do not involve an exposure aimed at reducing HIRRT will be excluded).

#### Comparators

Only studies that also included critically ill adult patients with AKI treated with RRT that did not receive the exposure or intervention, as a comparitor group, will be included. (Observational studies without an appropriate comparator will also be excluded).

#### Outcomes

- Primary outcome: HIRRT. Any definition used is acceptable provided that it is defined by the study’s methods. HIRRT encompasses not only changes in blood pressure (as in IDH) but also the initiation or increased dosing of vasopressors during RRT and/or any other criteria used to define hemodynamic instability during RRT (such as the need for fluid boluses). Studies that report HIRRT as an outcome but without any definition will be excluded.
- Secondary outcomes: death (any time points reported); ICU and hospital lengths of stay; renal recovery (dialysis independence at any time points reported); increased vasopressor use; need for interventions to treat HIRRT (e.g., fluid bolus, early stop of RRT session, reduction in ultrafiltration goal); cardiovascular events; system clotting; bleeding; dialysis adequacy; treatment-related symptoms (e.g., discomfort/cold/cramping).

### Search strategy

An initial search of MEDLINE and PubMed was performed to assess for prior systematic reviews on this subject. Prospero was searched for any currently registered systematic review on this topic. No prior or ongoing systematic review was identified.

In collaboration with other members of the research team, a health information specialist (LS) has constructed and will implement a comprehensive search strategy (refer to Appendix for sample search strategy).

As our search strategy, we will search the following databases:

- MEDLINE (1950–Feb 2016)
- EMBASE (1980–Feb 2016)
- CENTRAL (Cochrane Central Register of Controlled Trials)

We will supplement our search with a manual scan of bibliographies of all included studies. In addition, a search of the “gray literature” will be performed. This includes a search using “Google Scholar” and a scan of clinical trial registries for ongoing clinical studies (https://www.isrctn.com/). As well, this will include a review of abstracts from past 10 years from relevant scientific meetings:

- American Society of Nephrology Kidney Week
- Society for Critical Care Medicine (included in EMBASE search)
- International Symposium on Intensive Care and Emergency Medicine (included in EMBASE search)

Upon completion, eligible citations will be exported to a citation manager (EndNote, Thompson Reuters Industries, v. X7.3) for screening by two authors (AD, EC).

### Study selection

An initial screen of all identified abstracts will be conducted by two investigators (EC and AD) to determine whether the articles are eligible for further review. The articles considered for further review must have the following requirements:

- Original data from an original study (no review articles or editorials).
- Articles must specifically address HIRRT in patients with AKI.
- Any form of RRT for AKI is eligible except peritoneal dialysis.
- Only studies with titles published in English or French will be considered eligible for inclusion.
- Data from multiple reports assessing the same population or dataset will only be included once. If multiple reports are believed to represent the same population/dataset but report different outcomes or numbers of participants, then clarification will be sought from the corresponding author(s) regarding the totality of reporting for that population/dataset.

The same investigators will then conduct the full-text search to determine whether retrieved articles fulfill inclusion or exclusion criteria. Disagreements will be resolved by consensus where possible and by a third reviewer (SH) if not.

### Data extraction

Prior to duplicate extraction by two independent reviewers (EC, AD), a data extraction form will be created and piloted. The data extracted will include:

- Study characteristics: first author, year of publication, geographic location, setting (e.g., ICU, post-cardiac surgery), design (number of arms, crossover or not), duration of follow-up
- Sample characteristics: numbers of participants in each arm, age, sex, inclusion and exclusion criteria, baseline imbalances across study arms, other possible confounders
- Details regarding interventions and co-interventions: dose/type of intervention, blinding, dropouts, and study withdrawals
- Outcomes assessed: primary outcome of HIRRT vs other, definition of HIRRT and other outcomes assessed, description of measurement tools/devices.
- Effect of interventions/exposures: data describing categorical or continuous efficacy variables for the included outcomes, adverse events

Data will be entered into Review Manager (RevMan 5.3) by one reviewer (AD) with verification by the second reviewer (EC).

### Quality assessment

Two independent investigators (EC and AD) will assess the included studies for potential bias and the quality of reporting.

Observational cohort studies will be assessed using the Cochrane Risk of Bias Assessment Tool for Non-Randomized Studies of Interventions (ACROBAT-NRSI) [12]. Quality of reporting for observational studies will be assessed according to the Strengthening the Reporting of Observational Studies in Epidemiology (STROBE) checklist [13]. The Newcastle-Ottawa Scale (NOS) will be implemented to assess the quality of nonrandomized studies with respect to design, content, and ease of use [14]. Any randomized controlled trials (RCTs) will be evaluated using the Cochrane Handbook “Risk of Bias” assessment tool [15].

Any disagreements will be resolved by consensus or, if that is not possible, a third reviewer (SH).

### Measures of treatment effect

RevMan 5.3 will be used for all analyses. For continuous data, we will report the mean differences between groups as well as the 95% confidence intervals.

### Data synthesis

Data will only be pooled for studies of similar design and limited heterogeneity; however, we expect that significant heterogeneity in interventions, comparator populations, and outcomes will prevent meta-analysis. We plan to examine potential sources of clinical and methodological heterogeneity according to study design, patient population, and outcomes measured as well as intervention and comparator characteristics. Should it be possible to do so, statistical heterogeneity will be assessed using the chi-squared test and defined using the *I* ^2^ statistic with thresholds for interpretation defined in accordance with the Cochrane Handbook for Systematic Reviews of Interventions [15].

Should meta-analysis not be possible or appropriate, the data will be primarily interpreted into a narrative synthesis. This synthesis will be organized according to the intervention/exposure(s) used to attempt to reduce or prevent HIRRT. For each intervention/exposure, they may be subgrouped according to the type of RRT used (i.e., hemodialysis, CRRT, or SLED). Other groupings and subgroupings that may be considered include the following:

- Outcomes of interest
- Cause of AKI
- Indication for RRT
- Treatment setting (e.g., ICU, all hospital, post-operative)
- Methodological quality
- Publication data
- Extent of adjustment for confounding

When possible, data will be interpreted into a quantitative synthesis. Data from included studies will be pooled whenever possible to explore the effect of any identified intervention on the primary or secondary outcomes:

- Frequency of HIRRT
- Mortality
- ICU and hospital length of stay
- Renal recovery

Assessment of reporting biases will be performed using a funnel plot (the standard error of the intervention effect estimate relative to the study size) if there are more than 10 studies included for analysis [16].

### Assessing the quality of the evidence

An assessment of the quality of the evidence for the primary outcome from “very low” to “high” will be made in accordance with the criteria suggested by the Grading of Recommendations Assessment, Development and Evaluation (GRADE) Workgroup [17].

## Discussion

HIRRT is a common complication of renal replacement therapy not only in ESKD but also in the critically ill AKI population. The consequences of HIRRT can range from minor to serious events including bowel ischemia, myocardial infarction, and access thrombosis [2]. HIRRT can lead to further loss of residual renal function over time [5, 6]. Research suggests that HIRRT can also adversely impact renal recovery in critically ill patients with AKI [9]. To date, most studies on HIRRT and interventions to prevent it are conducted on maintenance hemodialysis patients with ESKD and thus do not necessarily apply to critically ill patients with AKI who require RRT.

This review proposes to use a systematic approach to identify, gather, and summarize the currently existing evidence on interventions to prevent HIRRT in critically ill patients with AKI on RRT from interventional and observational studies as well any ongoing trials. We aim to assess the effectiveness of strategies used to limit or prevent HIRRT in AKI by assessing specific, clinically important outcomes. In addition to exploring strategies to limit HIRRT in AKI, we will also review the available evidence for major outcomes associated with HIRRT, which includes mortality, ICU/hospital length of stay, and renal recovery. This summary of evidence can help guide clinical decisions regarding RRT parameters in AKI.

We expect that most of the evidence will come from observational studies rather than RCTs. As there is a higher risk of bias in observational studies, we have chosen to implement in our protocol three accepted assessment tools, the Cochrane ACROBAT-NRSI [10], the STROBE checklist [11], and the Newcastle-Ottawa Scale [12]. We will also use an accepted risk of bias assessment tool for any RCTs [13]. The information we obtain will be dependent on the quality of available evidence, and as such, there will likely be the need for cautious interpretation of the findings. The information from this systematic review will inform clinicians of the current evidence for interventions to limit or prevent HIRRT in AKI and will highlight areas in need of further study. We expect that our summary of evidence will be useful in formulating further research protocols to expand our knowledge in this area, which will ultimately serve to better care for critically ill AKI patients requiring RRT.

## Appendix

**Table 1:** Sample MEDLINE search strategy

1	exp Acute Kidney Injury/
2	(acute adj (kidney or renal or nephr* or tubular or dialys*)).ti.
3	(acute adj2 (kidney or renal)).tw.
4	((crescent* or progressive or anca* or acute) and (glomerul* or nephrit*)).ti.
5	((kidney or renal) adj isch?emi*).ti.
6	*Nephritis, Interstitial/ci [Chemically Induced]
7	*Hemorrhagic Fever with Renal Syndrome/
8	(induced adj (kidney injury or renal injury)).tw.
9	Oliguria/
10	acute nephr*.tw.
11	(pre-renal or prerenal).tw.
12	((arf or aki) and (renal or kidney)).tw.
13	((nephropath* and (contrast* adj (medi* or induced or agent*))) or radiocontrast* or iodinated or crystal* or cast).mp.
14	(nephrotox* or (renal and toxi*)).ti.
15	renal tubul*.ti.
16	14 or 15
17	ci.fs. or contrast medi*.tw. or induced.mp.
18	16 and 17
19	((kidney or renal) adj isch?emi*).tw.
20	Kidney Tubules, Proximal/
21	ur?emi*.ti.
22	renal inflammation.tw.
23	19 or 20 or 21 or 22
24	*Reperfusion Injury/
25	isch?emi* reperfusion.tw. or injury.ti. or acute.tw.
26	24 or 25
27	23 and 26
28	h?emolytic ur?emi*.ti.
29	(thrombotic adj (thrombocytopeni* or microangiopathy)).tw.
30	28 or 29
31	(kidney or renal or acute).mp.
32	30 and 31
33	(induced adj (kidney or renal)).tw.
34	(nephrotox* or contrast medi* or reperfusion or perfusion).mp.
35	33 and 34
36	((tubulointerstitial or interstitial or anti-glomerular or antiglomerular) and (glomerul* or nephrit*)).ti.
37	(acute or crescentic or atypical or progressive).mp.
38	36 and 37
39	1 or 2 or 3 or 4 or 5 or 6 or 7 or 8 or 9 or 10 or 11 or 12 or 13 or 18 or 27 or 32 or 35 or 38
40	exp Renal Replacement Therapy/
41	((kidney* or renal) adj3 replacement adj2 therap*).tw.
42	exp Renal Dialysis/
43	((renal or extracorporeal) adj2 dialys#s).tw.
44	exp Hemofiltration/
45	h?mofiltration*.tw.
46	h?modialys#s.tw.
47	h?modialfiltration*.tw.
48	Dialysis/
49	cavf.tw.
50	sustained low-efficiency dialysis.tw.
51	sled.tw.
52	or/40-51
53	hypotension/ or hypotension, orthostatic/
54	hypotens*.tw.
55	low blood pressure.tw.
56	vasopressor*.tw.
57	IDH.tw.
58	h?modynamic*.tw.
59	or/53-58
60	39 and 52 and 59
61	exp Adult/
62	adult.tw.
63	61 or 62
64	60 and 63

## Additional file

Additional file 1: PRISMA-P 2015 Checklist. (DOCX 36 kb)

## Abbreviations

ACROBAT-NRSI: Assessment Tool for Non-Randomized Studies of Interventions
AKI: Acute kidney injury
CRRT: Continuous renal replacement therapy
ESKD: End-stage kidney disease
GRADE: Grading of Recommendations Assessment, Development and Evaluation
HIRRT: Hemodynamic instability during renal replacement therapy
ICU: Intensive care unit
IDH: Intradialytic hypotension
NOS: Newcastle-Ottawa Scale
PRISMA: Preferred Reporting Items for Systematic Reviews and Meta-Analyses
PROSPERO: Prospective Register of Systematic Reviews
RCT: Randomized controlled trial
RRT: Renal replacement therapy
SLED: Slow low-efficiency dialysis
STROBE: Strengthening the Reporting of Observational Studies in Epidemiology
